# Serum levels of fat-soluble vitamins in children with attention deficit hyperactivity disorder and relationship with symptom subtypes

**DOI:** 10.3389/fpsyt.2025.1646885

**Published:** 2025-10-28

**Authors:** Mengqi Li, Hailin Gu, Yan Chen

**Affiliations:** Department of Child Health Care, Wuhan Children’s Hospital (Wuhan Maternal and Child Healthcare Hospital), Tongji Medical College, Huazhong University of Science & Technology, Wuhan, China

**Keywords:** attention deficit hyperactivity disorder, fat-soluble vitamins, vitamin A, vitamin D, vitamin E

## Abstract

**Objective:**

To evaluate the levels of vitamin A (VA), vitamin D (VD) and vitamin E (VE) in children with Attention deficit hyperactivity disorder(ADHD) and analyze their association with ADHD symptoms.

**Methods:**

A total of 657 children aged 4–10 years were collected, of whom 219 were diagnosed with ADHD (including 100 cases of attention deficit major, 14 cases of hyperactive impulsivity major, and 105 cases of mixed type) and 438 were used as healthy controls. High performance liquid chromatography (HPLC) was used to detect serum VA, VD (D2, D3 and total VD) and VE levels. The Weiss Functional Deficit Rating Scale (WFIRS) was used to evaluate the clinical symptoms of ADHD.

**Results:**

The serum levels of VA, VD (D2, D3, total VD) and VE in the ADHD group were significantly lower than those in the control group. Among the different subtypes, the levels of VD3, total VD and VA in the attention deficit type and mixed type were significantly different from those in the control group, and the levels of total VD and VA in the hyperactive and impulsive type were significantly different. There was a correlation between the total score of ADHD symptoms and the scores of each dimension and vitamin levels.

**Conclusion:**

The level of fat-soluble vitamins is significantly correlated with the prevalence, subtype symptoms and functional deficits of ADHD, suggesting that VA, VD and VE supplementation may be the adjuvant treatment for ADHD, but the specific causal relationship needs to be verified by further prospective studies.

## 1 Introduction

Attention deficit hyperactivity disorder (ADHD) is one of the most prevalent neurobehavioral disorders in childhood, characterized by age-inappropriate inattention and/or hyperactivity-impulsivity (1). It is classified into three clinical subtypes: predominantly inattentive type, predominantly hyperactive-impulsive type, and combined type. Although ADHD originates in childhood, its symptoms often persist into adolescence and adulthood, with some perspectives suggesting that ADHD may be a lifelong disorder. In recent years, the prevalence of ADHD has shown an upward trend. Global estimates indicate a prevalence rate of 7.6% (2), with some regions reporting rates as high as 10.3% (3). Notably, males exhibit a significantly higher prevalence than females. Research demonstrates that the onset of ADHD results from the combined action of genetic and environmental factors (4), yet its specific pathogenesis remains not fully understood. Existing studies have indicated that mutations in gene loci, vitamin and mineral supplementation, and gut microbiota levels are all associated with the development of ADHD and the severity of its symptoms (5–7).

In recent years, a substantial body of research has focused on the relationship between ADHD and nutritional elements, particularly fat-soluble vitamins such as vitamin A (VA), vitamin D (VD), and vitamin E (VE). VD, a neurosteroid hormone, is primarily synthesized from 7-dehydrocholesterol in the skin upon exposure to sunlight or obtained from dietary sources of VD2. By binding to VD receptors, VD exerts effects on various organs throughout the body. It regulates the expression of multiple genes, and its deficiency impacts the expression of synaptic proteins as well as the synthesis and metabolism of neurotransmitters. Furthermore, VD receptors are widely distributed in the brain, with particularly high concentrations in the substantia nigra, an area rich in dopaminergic neurons (8). Studies have demonstrated that VD functions as an enzyme involved in dopamine synthesis and enhances the expression of tyrosine kinase (9). Given that ADHD is closely associated with dysfunction of the dopamine transporter (DAT), which diminishes dopamine signaling by facilitating the reuptake of dopamine into presynaptic neurons, thereby influencing the cognitive function of ADHD patients (10), VD metabolism may play a role in the pathogenesis of ADHD.

On the other hand, VA is metabolized into retinoic acid (RA) within the body, where it is involved in hippocampal synaptic plasticity and plays a crucial role in memory that is dependent on the hippocampus (11). Animal studies have demonstrated that an increase in RA concentration is directly associated with improved memory performance (12). Additionally, human studies have indicated that lower circulating levels of retinol are predictive of a higher risk of cognitive function decline (13). Therefore, a deficiency in VA may impair hippocampal synaptic plasticity, leading to a decline in memory function, insufficient learning ability, and attention maintenance disorders, which may exacerbate cognitive deficits in patients with ADHD.

VE, primarily in the form of α-tocopherol, serves as a significant fat-soluble antioxidant and a crucial component of cellular membranes (14). VE effectively scavenges peroxyl radicals, halting the oxidation of polyunsaturated fatty acids (PUFAs) and thereby preventing oxidative damage. Furthermore, VE plays a role in modulating the immune system, mitigating inflammatory responses, and alleviating various types of stress (15). Research indicates that VE can reduce oxidative stress and neuroinflammation in patients with ADHD through its antioxidant, anti-inflammatory, and neuronal membrane-protective properties. This action contributes to improvements in hyperactivity, impulsivity, and overall functional status. Conversely, VE deficiency may worsen neuronal damage and inflammatory responses, potentially exacerbating ADHD symptoms.

Current research on the impact of fat-soluble vitamins on ADHD remains contentious and incomplete. Some preliminary studies have indicated a potential association between deficiencies in VD and VA and the occurrence of ADHD (16). However, other studies have found no significant correlation between VD levels and ADHD (17). To date, no research has established a relationship between VE levels and ADHD. In light of this background, the present study aims to evaluate the levels of VD, VA, and VE in children diagnosed with ADHD and to investigate their correlations with ADHD symptoms.

## 2 Participants and methods

### 2.1 Participants

We collected patient information from 657 children aged 4 to 10 years who visited Wuhan Children’s Hospital due to “inattention or hyperactivity” between June 2023 and June 2024. All children suspected of having ADHD underwent a comprehensive evaluation, which included a detailed review of their current health status, developmental history, and family medical history. Developmental pediatricians conducted thorough physical examinations and in-depth interviews with parents in accordance with the diagnostic criteria outlined in the *Diagnostic and Statistical Manual of Mental Disorders* (DSM-5). This approach aimed to gain a comprehensive understanding of the children’s daily performance and behavioral characteristics, thereby facilitating accurate ADHD diagnoses. Ultimately, 219 children met the diagnostic criteria for ADHD, comprising 171 boys and 48 girls, with an average age of 8.15 years. Among these, 100 cases were classified as predominantly inattentive type, primarily characterized by difficulties in maintaining attention, easy distraction by external stimuli, and frequent lapses in focus during academic tasks and other activities requiring concentration. Additionally, 14 cases were identified as predominantly hyperactive-impulsive type, exhibiting behavioral characteristics such as excessive limb movements, an inability to remain seated quietly, challenges in participating in activities quietly, and often accompanied by excessive running and noisy behaviors. Furthermore, 105 cases were classified as combined type, exhibiting core symptoms of both inattention and hyperactivity-impulsivity, with more complex and diverse clinical manifestations. Simultaneously, 438 healthy children without any physical or behavioral abnormalities were selected from the same outpatient clinic to serve as the healthy control (HC) group, which included 340 boys and 98 girls, with an average age of 7.93 years.This study was approved by the Medical Ethics Committee of Wuhan Children’s Hospital Affiliated with Huazhong University of Science and Technology (No. 2025592).

### 2.2 Exclusion criteria

The exclusion criteria were children: (1) Children with other genetic, neuropsychiatric and physical diseases, such as cerebral palsy, epilepsy, and schizophrenia; (2) Have not taken nutritional supplements containing vitamins such as VA, VD, VE and so on in the past 3 months; (3) Children suffering from malnutrition, picky eating, digestive diseases, etc., which may affect the absorption and metabolism of micronutrients. (4) Taking drugs that may affect the metabolism of VA, VD or VE.

### 2.3 Behavioral assessments

The Weiss Functional Impairment Rating Scales (WFIRS) was developed by Margaret D Weiss in 2007 to assess the impact of symptoms and behavioral or emotional problems on clinically relevant functional domains in children with ADHD. WFIRS is divided into self-report and parent versions. The Weiss Functional Impairment Rating Scale-Parent Report (WFIRS-P) was assessed by parents or caregivers, and the Chinese version was revised in 2001 by the team of Professor Wang Yufeng, Institute of Mental Health, Peking University. WFIRS – P consists of a total of 50 items divided into 6 functional areas, namely family, learning and school, life skills, children’s self-concept, social activities and risk-taking activities. Each item is scored on a four-point Likert scale: 0 (never), 1 (sometimes), 2 (often), and 3 (always or often). This study used the Chinese version of the WFIRS-P scale.

### 2.4 Laboratory measurements

High-performance liquid chromatography was employed to conduct blood tests on all enrolled children, assessing various detection indices, including the levels of vitamin A (VA), vitamin D2 (VD2), vitamin D3 (VD3), total vitamin D (VD), and vitamin E (VE). Serum VA levels < 300 ng/ml and 300–800 ng/ml were judged to be VA deficient and normal, respectively. Serum VD levels < 30 ng/ml and 30–100 ng/ml were judged to be VD insufficiency/deficiency and normal, respectively. SerumVE levels < 5168.6 ng/ml, 5168.6–20000 ng/ml and >20000 ng/ml were judged to be VE deficiency, normal and overdose, respectively.

### 2.5 Statistical analysis

All data analyses were conducted using SPSS version 27.0. Prior to analysis, the Kolmogorov-Smirnov test was utilized to evaluate the normality of continuous variables. Continuous data are presented as mean ± standard deviation, while categorical data are expressed as frequencies and percentages. Based on the distribution of the variables, independent samples t-tests were employed to compare continuous data between the two groups. Chi-square tests were utilized to identify differences in categorical variables between the groups. The Spearman correlation coefficient test was applied to assess the significance of factors influencing serum concentrations of fat-soluble vitamins. A p-value of less than 0.05 was considered statistically significant.

## 3 Results

### 3.1 Comparison of sociodemographic features between the HC group and ADHD group

**Table 1** shows the sociodemographic data of the two groups. There were no significant differences in gender or age between children with ADHD and those in the HC (healthy control) group.

**Table 1:** Demographic characteristics.

Characteristic	ADHD	Control	t/χ^2^	*P*
Number of participants	219	438		
Age, mean (SD), year	8.15(1.76)	7.93(3.41)	1.108	0.268
Sex, *n* (%)			0.018	0.894
Male	171(78.08)	340(77.63)		
Female	48(21.92)	98(22.37)		

### 3.2 Comparison of vitamin content between the case and HC groups

Serum concentrations of VA, VD, and VE in the two groups are shown in **Table 2**. The data show that in terms of VD3, VD2, VD, VA, and VE. The mean contents in the ADHD group were 22.54 ng/ml, 2.03 ng/ml, 24.58 ng/ml, 330.39 ng/ml, and 9722.09 ng/ml, respectively, all lower than the control group’s corresponding values of 27.08 ng/ml, 3.31 ng/ml, 30.38 ng/ml, 376.04 ng/ml, and 10440.40 ng/ml. The mean contents of VD3, VD2, VD, VA, and VE in the ADHD group were all lower than those in the control group. Results of independent samples t-tests showed t-values of -7.692, -3.563, -8.886, -7.249, and -3.651, respectively, with all P < 0.001, indicating extremely significant differences.

**Table 2:** Comparison of vitamin content between the case and HC groups.

Vitamin, mean (SD), ng/ml	ADHD	Control	t	*P*
D3	22.54(7.02)	27.08(7.17)	-7.692	<0.001
D2	2.03(3.23)	3.31(5.92)	-3.563	<0.001
D	24.58(7.34)	30.38(8.15)	-8.886	<0.001
A	330.39 (76.84)	376.04 (75.71)	-7.249	<0.001
E	9722.09 (2271.72)	10440.40 (2428.51)	-3.651	<0.001

### 3.3 Comparison of vitamins between different ADHD types and the control group

**Table 3** compares the vitamin contents of different ADHD subtypes (inattentive type, hyperactive-impulsive type, combined type) with those of the control group: In terms of VD3, the contents in all ADHD subtypes were significantly lower than those in the control group (*P* < 0.001 for the inattentive and combined types, and *P* = 0.002 for the hyperactive-impulsive type). For VD2, only the combined type showed an extremely significant decrease compared to the control group (*P* < 0.001), with no significant differences in the other subtypes. VD levels in all subtypes were significantly lower than those in the control group (*P* < 0.001 for the inattentive and combined types, and *P* = 0.001 for the hyperactive-impulsive type). VA contents in all subtypes were significantly lower than those in the control group (*P* < 0.001). For VE, the inattentive and combined types were significantly lower than the control group (*P* = 0.001 and *P* = 0.038, respectively), while there was no significant difference in the hyperactive-impulsive type (*P* = 0.160).

**Table 3:** Comparison of vitamin between different ADHD types and control group.

ADHD types/control	D3	D2	D	A	E
Control	27.08 (7.17)	3.31 (5.92)	30.38 (8.15)	376.04 (75.71)	10440.40 (2428.51)
inattentive type	21.64 (6.80)	2.28 (4.14)	23.92 (7.46)	337.71 (69.36)	9574.08 (2050.67)
t	-6.904	-1.651	-7.264	-4.637	-3.308
*P*	<0.001	0.099	<0.001	<0.001	0.001
hyperactive-impulsive type	21.13 (5.22)	2.06 (1.73)	23.19 (5.27)	308.16 (67.78)	9515.51 (2202.92)
t	-3.076	-0.788	-3.278	-3.312	-1.406
*P*	0.002	0.431	0.001	0.001	0.160
combined type	23.59 (7.32)	1.80 (2.26)	25.39 (7.42)	326.38 (84.20)	9890.59 (2480.41)
t	-4.453	-4.204	-5.726	-5.903	-2.075
*P*	<0.001	<0.001	<0.001	<0.001	0.038

### 3.4 Relationship between total ADHD deficit scores and vitamin levels

**Table 4** uses Spearman correlation analysis to explore the relationship between total ADHD deficit scores and vitamin levels. The results show VD3 was significantly negatively correlated with the total score, family dimension, and learning and school dimension (*P* < 0.05). VD2 was only significantly positively correlated with the self-management dimension (*P* < 0.05). VD had no significant correlation with any dimension (*P* > 0.05). VA was only significantly positively correlated with the learning and school dimension (*P* < 0.05). VE was significantly negatively correlated with the total score, family dimension, life skills dimension, self-management dimension, and social activities dimension (*P* < 0.05). To further clarify the predictive effect of each vitamin level on ADHD functional deficit scores, we conducted a multiple linear regression analysis, the results of which are presented in **Table 5**. The results showed that among all vitamin indices and functional deficit dimensions: VA exhibited a significant positive predictive effect on the learning and school dimension score (β=5.279, P=0.001) and a significant negative predictive effect on the social dimension score (β=-4.491, P=0.029), suggesting that it may simultaneously affect learning-related functional impairments and social function improvement. VE showed a significant negative predictive effect on the social dimension score (β=-133.325, P=0.031), supporting its potential role in alleviating social functional deficits.VD3, VD2, and VD levels had no significant predictive effects on any dimension scores (all P>0.05); VE also had no significant predictive effects on the total score, family, life skills, and other dimensions, while VA showed no significant predictive effects on non-learning and non-social dimensions (all P>0.05).

**Table 4:** Relationship between total ADHD deficit scores and vitamin levels.

functional deficit dimensions	D3			D2		D		A		E	
	*r*	*P*	*r*	*P*	*r*	*P*	*r*	*P*	*r*	*P*
Total Score	0.000	0.996	0.041	0.550	-0.001	0.985	0.099	0.142	-0.197	0.003*
Family	0.002	0.982	0.047	0.485	0.007	0.917	0.044	0.514	-0.158	0.019*
Learning and school	0.001	0.992	-0.046	0.495	-0.032	0.634	0.220	0.001*	-0.056	0.412
Life skills	0.058	0.392	0.006	0.935	0.058	0.393	0.038	0.571	-0.163	0.016*
Self-management	-0.06	0.378	0.146	0.031*	-0.017	0.807	0.112	0.097	-0.135	0.046*
Social	-0.086	0.207	0.064	0.349	-0.084	0.213	-0.029	0.668	-0.179	0.008*
Adventure activities	0.022	0.747	0.019	0.781	0.021	0.761	0.012	0.854	-0.127	0.061

Spearman rank correlation analysis was used to analyze the correlation between each dimension of ADHD deficiency scores and vitamin levels. ADHD, attention deficit hyperactivity disorder; r, correlation coefficient. * there is a statistical difference between the two groups, p< 0.05.

**Table 5:** Linear regression analysis between vitamin levels and ADHD deficiency scores.

functional deficit dimensions	D3		D2		D		A		E	
	*β*	*P*	*β*	*P*	*β*	*P*	*β*	*P*	*β*	*P*
constant	21.820	<0.001	2.679	<0.001	24.499	<0.001	304.937	<0.001	10161.200	<0.001
Family	-0.021	0.894	-0.014	0.848	-0.035	0.832	0.514	0.764	65.473	0.204
Learning and school	0.090	0.552	-0.104	0.132	-0.014	0.927	5.279	0.001	28.064	0.563
Life skills	0.188	0.193	-0.004	0.953	0.184	0.222	0.709	0.644	-19.719	0.669
Self-management	0.019	0.951	0.210	0.148	0.230	0.487	3.346	0.322	8.726	0.932
Social	-0.207	0.281	-0.028	0.748	-0.235	0.241	-4.491	0.029	-133.325	0.031
Adventure activities	-0.111	0.670	-0.021	0.862	-0.132	0.628	-1.052	0.705	-55.019	0.510

Multiple linear regression was used to analyze the relationship between vitamin levels and ADHD deficiency scores.

ADHD, attention deficit hyperactivity disorder; β, regression coefficient.

## 4 Discussion

This study yielded three significant findings. First, there were notable differences in vitamin content between the ADHD group and the control group, with levels of vitamins A, D (including D3 and D2), and E being significantly lower in the ADHD group. This suggests a potential association between vitamin levels and ADHD. Second, specific ADHD subtypes exhibited distinct relationships with vitamin levels. The findings indicated that, relative to the control group, various ADHD subtypes demonstrated significant differences in multiple vitamin contents. Notably, the inattentive type and the combined type showed significant differences in levels of VD3, D, and A, while the hyperactive-impulsive type exhibited significant differences in VD and A levels. These results imply that certain ADHD subtypes may correlate with vitamin levels; however, the causal relationship remains to be confirmed through further research. Third, ADHD deficit scores displayed multidimensional correlations with vitamin levels. The study revealed that total ADHD deficit scores were correlated with vitamin levels to varying extents. Specifically, VD2 was positively correlated with the self-management dimension, VA was positively correlated with the learning and school dimension, and VE was negatively correlated with the total score and dimensions such as family, learning and school, life skills, self-management, social activities, and risk-taking activities. These findings highlight the varying associations between different vitamins and the dimensions of ADHD, illuminating the complex relationship between vitamin levels and ADHD deficit scores, and providing a crucial direction for future research on their association.

In recent years, numerous studies have demonstrated that VD plays a significant role in brain development and function, influencing neurotransmission, neuroimmunomodulation, and exhibiting antioxidant effects. Furthermore, VD is implicated in behavioral and neuropsychiatric disorders (18, 19). Epidemiological evidence from previous meta-analyses indicates that insufficient perinatal VD levels substantially increase the risk of developing attention-deficit/hyperactivity disorder (ADHD) later in life (20). Additionally, VD supplementation has been shown to alleviate ADHD symptoms without causing severe adverse reactions (21). Our study found that VD levels were significantly lower in children with ADHD, aligning with the conclusions of the aforementioned studies. From a neurobiological perspective, dopaminergic dysfunction in the prefrontal cortex represents a critical link in the pathogenesis of ADHD (22), and VD is essential for the development and functional maintenance of dopaminergic neurons. Animal studies have demonstrated that VD deficiency during developmental stages leads to alterations in brain morphology and structure, disrupts the normal development of dopaminergic pathways, and results in dysfunction across multiple neurotransmitter systems (22). VD regulates the genetic expression of tyrosine hydroxylase, a rate-limiting enzyme in dopamine synthesis, thereby modulating dopamine biosynthesis (9). Furthermore, VD influences the synthesis of glial cell line-derived neurotrophic factor (GDNF) and its receptor, which modulates the survival and differentiation of dopaminergic neurons and impacts the GDNF/Ret signaling pathway, thereby maintaining the normal function of dopaminergic neurons (23). These neurodevelopmental abnormalities may constitute the neurobiological basis for the occurrence of ADHD. As an immunomodulatory molecule, VD is associated with ADHD and its related immune-mediated diseases. Multiple studies have focused on the relationship between cytokines and ADHD, revealing the potential role of inflammatory responses in the neurodevelopmental mechanisms underlying ADHD. Two independent studies have demonstrated that interleukin-6 (IL-6) is the only cytokine exhibiting a significant upward trend in ADHD patients (24, 25). Additionally, research on children with ADHD has indicated that plasma levels of IL-16 and IL-13 correlate with executive function performance (26). Our study, along with previous data, suggests that VD levels may influence the risk of developing ADHD and executive function by affecting brain development and immune-inflammatory responses. Therefore, monitoring and supplementation of VD should be considered fundamental interventions for children with ADHD.

Our findings showed that VA levels in the patient group were significantly lower than in the control group, and that VA levels were positively correlated with school-related behavioral problems. VA and its active metabolite retinoic acid (RA) may be linked with ADHD and retinal development through an association with the nervous system. Retinoic acid is believed to maintain the function of the central nervous system and hippocampal synaptic plasticity. VA deficiency impairs hippocampal synaptic plasticity, diminishing learning and attention (27). Any additional deficiency of VA will lead to abnormal development of neural pathways of the Central Nervous System thereby increasing the risk of attention deficit hyperactivity. Studies on animals have shown that prenatal models of ADHD in mice have synaptic abnormalities in the hippocampus (28). Neuroimaging of hyperactivity disorder patients showed reduced intracranial and hippocampal volumes (29). The behavioral issues of individuals with ADHD may correspond with an inability of their synapses to change. As reported by researchers, RA regulates various actions in the nervous system, dopaminergic signalling pathways and also helps in neurogenesis and differentiation of striatal neurons which may cause disordered behavior (30). Prior research has suggested that VA and RA supplementation is able to improve motor-related problems (31, 32), which is consistent with our findings. Moreover, a study has shown that VA supplementation reduces the serotonin level (33). Additionally, both dopamine and serotonin are involved in the pathophysiology of ADHD (34) To conclude, VA and its metabolites have different effects on ADHD pathogenesis and behavioral functions.

In this study, we found that VE levels were associated with the prevalence of ADHD and various behavioral deficits. As a fat-soluble organic antioxidant, VE protects cell membranes and nucleic acids, reduces reactive oxygen species (ROS), and lipid peroxidation products such as malondialdehyde (MDA), thereby mitigating oxidative stress damage to cells (35). Furthermore, VE serves as an effective immunomodulator present in the cell membranes of all cells, safeguarding them from oxidative damage, preserving the oxidation of polyunsaturated fatty acids in immune cell membranes, and decreasing the production of inflammatory markers such as tumor necrosis factor (TNF)-α and interleukin-6 (IL-6) (36). Existing studies have identified a significant positive correlation between IL-6, tumor necrosis factor, and hyperactive-impulsive ADHD in children and adolescents (37), which aligns with our findings. Additionally, the immunomodulatory role of VD is also linked to IL-6, leading us to speculate that VD and VE may have a certain association in their immunological effects on ADHD. Multiple prior animal studies have demonstrated a clear connection between VE deficiency and impaired immune function, with VE supplementation capable of reversing this impairment (38, 39). ADHD is associated with immunoregulatory defects, and abnormal immune function may play a role in the pathological process of ADHD. Thus, immune dysfunction resulting from VE deficiency may increase the risk of developing ADHD, while maintaining adequate VE levels could be crucial for sustaining normal immune function and preventing ADHD.

## 5 Conclusion

In conclusion, the levels of fat-soluble vitamins are significantly associated with the prevalence of ADHD, its subtype symptoms, and functional deficits. This finding opens new avenues for investigating the pathological mechanisms underlying ADHD and for developing clinical interventions. For ADHD patients, particularly those with vitamin deficiencies, supplementation with vitamins A, D, and E may serve as an effective adjuvant therapy. Furthermore, the establishment of tailored vitamin monitoring and supplementation protocols based on ADHD subtypes merits further investigation. However, the specific causal relationships remain unclear, necessitating additional prospective studies and intervention trials to confirm the impact of vitamin supplementation on ADHD.

### 5.1 Limitations

This study has several limitations. Firstly, it employs a cross-sectional survey design with a case-control analysis, which restricts the ability to infer the duration of fat-soluble vitamin deficiencies from the collected data. Consequently, we can only establish correlations rather than infer causal relationships. Secondly, the levels of fat-soluble vitamins are influenced by various factors, including familial influences, dietary behaviors, sunlight exposure duration, and the timing of blood sampling. Unfortunately, we were unable to analyze the impacts of these factors.Third, this study is a cross-sectional study, and the effect on long-term vitamin deficiency cannot be assessed. In future studies, longitudinal studies can be designed to track the relationship between vitamin deficiency and the development of ADHD symptoms, and to investigate and control factors that may affect vitamin levels, such as diet and lifestyle habits, in more detail when collecting samples.

## Data Availability

The raw data supporting the conclusions of this article will be made available by the authors, without undue reservation.
